# Complete Genome Sequence of the Polysaccharide-Degrading Rumen Bacterium *Pseudobutyrivibrio xylanivorans* MA3014 Reveals an Incomplete Glycolytic Pathway

**DOI:** 10.1093/gbe/evaa165

**Published:** 2020-08-08

**Authors:** Nikola Palevich, Paul H Maclean, William J Kelly, Sinead C Leahy, Jasna Rakonjac, Graeme T Attwood

**Affiliations:** e1 AgResearch Limited, Grasslands Research Centre, Palmerston North, New Zealand; e2 Institute of Fundamental Sciences, Massey University, Palmerston North, New Zealand; e3 Donvis Limited, Palmerston North, New Zealand

**Keywords:** *Pseudobutyrivibrio xylanivorans*, genome, rumen, bacteria, polysaccharide, enolase

## Abstract

Bacterial species belonging to the genus *Pseudobutyrivibrio* are important members of the rumen microbiome contributing to the degradation of complex plant polysaccharides. *Pseudobutyrivibrio xylanivorans* MA3014 was selected for genome sequencing to examine its ability to breakdown and utilize plant polysaccharides. The complete genome sequence of MA3014 is 3.58 Mb, consists of three replicons (a chromosome, chromid, and plasmid), has an overall G + C content of 39.6%, and encodes 3,265 putative protein-coding genes (CDS). Comparative pan-genomic analysis of all cultivated and currently available *P. xylanivorans* genomes has revealed a strong correlation of orthologous genes within this rumen bacterial species. MA3014 is metabolically versatile and capable of growing on a range of simple mono- or oligosaccharides derived from complex plant polysaccharides such as pectins, mannans, starch, and hemicelluloses, with lactate, butyrate, and formate as the principal fermentation end products. The genes encoding these metabolic pathways have been identified and MA3014 is predicted to encode an extensive range of Carbohydrate-Active enZYmes with 78 glycoside hydrolases, 13 carbohydrate esterases, and 54 glycosyl transferases, suggesting an important role in solubilization of plant matter in the rumen.

**Significance**

In this article, we report the first high-quality and closed multireplicon genome of the rumen bacterium *Pseudobutyrivibrio xylanivorans* MA3014. Furthermore, we provide phenotypic characterization and comparative genotypic analyses with other rumen *Pseudobutyrivibrio xylanivorans*, a group of organisms with important roles in ruminal xylan and pectin breakdown. These analyses show that rumen *Pseudobutyrivibrio xylanivorans* encode a large and diverse spectrum of degradative carbohydrate-active enzymes (CAZymes) and binding proteins involved in the depolymerization and utilization of insoluble plant polysaccharides. The reconstruction of their metabolic pathways combined with metabolite analysis provides a deeper insight into their metabolism and adaptation to life in the rumen.

## Introduction

*Pseudobutyrivibrio* [family Lachnospiraceae, order Clostridiales] are anaerobic, monotrichous, butyrate-producing, curved rods, and have been isolated from or detected in the gastrointestinal tracts of various ruminants, monogastric animals, and humans (Kopečný et al. 2003; Willems and Collins 2009; Henderson et al. 2015). *Pseudobutyrivibrio* are among a small number of rumen genera capable of utilizing the complex plant structural polysaccharide xylan (Bryant and Small 1956; Hungate 1966). Two species of *Pseudobutyrivibrio* are currently recognized; *Pseudobutyrivibrio ruminis* and *Pseudobutyrivibrio xylanivorans* (Van Gylswyk et al. 1996; Kopečný et al. 2003). *Pseudobutyrivibrio xylanivorans* is commonly found in domestic and wild ruminants and the type strain Mz 5^T^ (DSM 14809) (Kopečný et al. 2003; Henderson et al. 2015) is able to utilize xylan, hemicellulose, and various oligo- and monosaccharides as substrates for growth (Zorec et al. 2000). Gaining an insight into the role of these microbial primary plant polysaccharide fermenters is important for understanding rumen function. Here, we present the complete genome sequence of *P. xylanivorans* MA3014, a strain isolated from a New Zealand pasture-grazed dairy cow (Noel 2013; Seshadri et al. 2018), and describe its comparison with other representative *P. xylanivorans* genomes.

## Materials and Methods

### Growth Conditions and Fermentation End Product Analysis

*Pseudobutyrivibrio xylanivorans* MA3014 was isolated from the rumen contents of fistulated Friesian dairy cattle and sequenced (Noel 2013; Seshadri et al. 2018). MA3014 was grown in RM02 medium (Kenters et al. 2011) with 10 mM glucose and 0.1% yeast extract but without rumen fluid and culture purity was confirmed by Gram stain. The morphological features of MA3014 cells were determined by both scanning and transmission electron microscopy of cells grown on RM02 medium alone or with the addition of neutral detergent fraction of plant material as previously described (Palevich et al. 2017,  Palevich, Kelly, Leahy, et al. 2019).

Growth on soluble substrates was assessed as an increase in culture density OD_600nm_ compared with cultures without carbon source added (all tested at 0.5% w/v final concentration), whereas total VFA production was used as an indicator of substrate utilization and growth for insoluble polymers (supplementary table S2, Supplementary Material online). VFA production was determined from triplicate broth cultures grown overnight in RM02 medium with cellobiose as substrate and analyzed for formate, acetate, propionate, n-butyrate, iso-valerate, and lactate on a HP 6890 series GC (Hewlett Packard) with 2-ethylbutyric acid (Sigma-Aldrich, St. Louis, MO) as the internal standard. To derivatize formic, lactic, and succinic acids, samples were mixed with HCl ACS reagent (Sigma-Aldrich, St. Louis, MO) and diethyl ether, with the addition of *N*-methyl-*N*-*t*-butyldimethylsilyltri-fluoroacetamide (MTBSTFA) (Sigma-Aldrich, St. Louis, MO) (Richardson et al. 1989).

### Preparation of Genomic DNA for Whole-Genome Sequencing

Genomic DNA was extracted from freshly grown cells by a modification of the standard cell lysis method previously described (Palevich, Kelly, Leahy, et al. 2019; Seshadri et al. 2018), followed by phenol–chloroform extraction, and purification using the Qiagen Genomic-Tip 500 Maxi Kit (Qiagen, Hilden, Germany). Specificity of genomic DNA was verified by automated Sanger sequencing of the 16*S* rRNA gene following PCR amplification from genomic DNA. Total DNA amounts were determined using a NanoDrop ND-1000 (Thermo Scientific Inc.) and a Qubit Fluorometer dsDNA BR Kit (Invitrogen), in accordance with the manufacturer’s instructions. Genomic DNA integrity was verified by agarose gel electrophoresis and using a 2000 BioAnalyzer (Agilent).

### Genome Sequencing, Assembly, and Comparison

*Pseudobutyrivibrio xylanivorans* MA3014 was selected for genome sequencing as a NZ strain and only representative member of *P. xylanivorans* from the Hungate1000 collection (Seshadri et al. 2018; supplementary table S1, Supplementary Material online). The complete genome sequence of MA3014 was determined by pyrosequencing 3-kb mate paired-end (PE) sequence libraries using the 454 GS FLX platform with Titanium chemistry (Macrogen, Korea). Pyrosequencing reads provided 55× coverage of the genome and were assembled using the Newbler assembler (version 2.7, Roche 454 Life Sciences) which resulted in 116 contigs across 13 scaffolds. Gap closure was managed using the Staden package (Staden et al. 1999) and gaps were closed using additional Sanger sequencing by standard and inverse PCR techniques. In addition, MA3014 genomic DNA was sequenced using shotgun sequencing of 2-kb PE sequence libraries using the Illumina MiSeq platform (Macrogen, Korea) which provided 677-fold sequencing coverage. A de novo assembly was performed using the assemblers Velvet version 3.0 (Zerbino and Birney 2008), and EDENA version 3.120926 (Hernandez et al. 2008). The resulting sequences were combined with the Newbler assembly using the Staden package and Geneious, version 8.1 (Kearse et al. 2012). Genome assembly was confirmed by pulsed-field gel electrophoresis (Palevich 2011; Palevich, Kelly, Leahy, et al. 2019) and genome annotation was performed as described previously (Kelly et al. 2010). Genome comparisons of orthologous gene clusters within *Pseudobutyrivibrio* genomes were performed using OrthoVenn version 2 (Wang et al. 2015).

## Results and Discussion

### Genome Assembly, Properties, and Annotation

The genome of *P. xylanivorans* MA3014 was sequenced using short-read 454 GS FLX Titanium and Illumina technologies which generated 9.9 million PE reads (table 1). The MA3014 assembly with high coverage of 677× was achieved using insert sizes that ranged between 238 bp (Illumina MiSeq) and 2.5 kb (454 GS-FLX Titanium). In total, 2.6 Gb of trimmed and filtered sequence data were retained for the reported assembly. The assembled, closed genome is 3,584,491 bp with an overall %G + C content of 39.6% and consists of three replicons (Palevich 2011, 2016); a single chromosome (3,412,851 bp, %G + C 39.7), a chromid (PxyII, 88,942 bp, %G + C 36.9), and a plasmid (pNP95, 82,698 bp, %G + C 37.4). The overall genome statistics of MA3014 are similar to those from *P. xylanivorans* Mz 5^T^ (DSM 14809) and NCFB 2399 (DSM 10317) (Kopečný et al. 2003), are detailed in table 1. Gene prediction from the MA3014 genome sequence resulted in a total of 3,365 genes annotated of which 3,265 (97.03%) were CDS, and 81 were various RNA genes such as 16*S*/23*S*/tRNAs and so on (table 1). Putative functions were assigned to 2,364 (70.25%), whereas 901 CDS were annotated as hypothetical proteins or proteins of unknown function. In total, 840 (24.96%) genes have homology to proteins in the KEGG database, whereas 2,506 (74.47%) and 2,593 (77.06%) of annotated genes have well-defined PFAM and InterPro protein domains, respectively. In contrast, 153 (4.55%) of the annotated genes have identified type I signal peptide protein domain hits and are predicted to have extracellular functions. The MA3014 chromosome encodes 3,098 CDS, whereas the PxyII and pNP95 encode 96 and 71 genes, respectively. Overall, the coding region comprises 89.77% of the genome, which is typical of rumen *Butyrivibrio* and *Pseudobutyrivibrio* genomes sequenced within the Hungate1000 project (Seshadri et al. 2018; Palevich, Kelly, Leahy, et al. 2019).

**Table 1:** Comparison of Assembly and Annotation Statistics for the *Pseudobutyrivibrio xylanivorans* MA3014, Mz 5^T^, and NCFB 2399 Genomes

	MA3014		Mz 5^T^ (DSM 14809)		NCFB 2399 (DSM 10317)	
	Value	% of Total^a^	Value	% of Total^a^	Value	% of Total^a^
Genome project information						
Status	Complete		Draft		Draft	
Isolation source	Bovine rumen		Bovine rumen		Ovine rumen	
BioSample ID	SAMN12605118		SAMN02745725		SAMN02910350	
BioProject ID	PRJNA560993		PRJNA245713		PRJNA254867	
Assembly method	Newbler v. 2.3, Velvet v. 3.0, EDENA v. 3.1		vpAllpaths v. r46652		vpAllpaths v. r46652	
Genome coverage	677×		472×		402×	
Sequencing technology	454 GS-FLX Titanium, Illumina MiSeq		Illumina HiSeq 2000, HiSeq 2500		Illumina HiSeq 2000, HiSeq 2500	
Genome statistics						
Genome size (bp)	3,584,491	100	3,420,924	100	3,213,944	100
DNA coding (bp)	3,217,653	89.77	3,122,173	91.27	2,947,505	91.71
DNA G+C (bp)	1,417,916	39.56	1,324,549	38.72	1,272,039	39.58
DNA replicons/scaffolds	3	100	57	100	34	100
Genome annotations						
Total genes	3,365	100	3,150	100	2,965	100
Protein-coding genes	3,265	97.03	3,078	97.71	2,890	97.47
RNA genes	81	2.41	72	2.29	75	2.53
rRNA operons	3	—	2	—	4	—
tRNA genes	53	1.58	55	1.75	49	1.65
Genes in internal clusters	689	20.48	329	10.44	248	8.36
Genes with function prediction	2,364	70.25	2,397	76.1	2,275	76.73
Genes with enzymes	733	21.78	761	24.16	739	24.92
Genes connected to KEGG pathways	840	24.96	879	27.9	858	28.94
Genes connected to KEGG Orthology (KO)	1,433	42.59	1,486	47.17	1,460	49.24
Genes assigned to COGs	2,404	71.44	1,933	61.37	1,883	63.51
Genes with Pfam domains	2,506	74.47	2,502	79.43	2,367	79.83
Genes with TIGRfam domains	850	25.26	894	28.38	883	29.78
Genes with InterPro domains	2,593	77.06	1,702	54.03	1,608	54.23
Genes with signal peptides	153	4.55	180	5.71	167	5.63
Genes with transmembrane helices	818	24.31	837	26.57	816	27.52
CRISPR repeats	1	—	NA	—	1	—
Reference	This report		Kopečný et al. (2003)			

a The total is based on either the size of the genome in base pairs or the total number of genes or CDS in the annotated genome.

### Genome Comparison

A comparison of the *P. xylanivorans* MA3014 genome with the draft genomes of *P. xylanivorans* Mz 5^T^ (DSM 14809) and NCFB 2399 (DSM 10317) (Kopečný et al. 2003) is shown in table 1. The MA3014 genome is larger than both Mz 5^T^ and NCFB 2399, containing 187 and 375 more CDS, respectively. A feature of MA3014 is the presence of a chromid or secondary chromosome which is also found in other well-characterized *Butyrivibrio* genomes (Kelly et al. 2010; Palevich, Kelly, Ganesh, et al. 2019). Chromids are replicons with %G + C content similar to that of their main chromosome, but have plasmid-type maintenance and replication systems, are usually smaller than the chromosome (but larger than plasmids) and contain genes essential for growth along with several core genus-specific genes (Harrison et al. 2010). The PxyII replicon has been designated as a chromid of MA3014 as it possesses all of these characteristics and contains genes encoding enzymes that have a role in carbohydrate metabolism and transport. Since the PxyII chromid is 2,834 bp smaller than the Bhu II chromid of *Butyrivibrio hungatei* MB2003, it is now the smallest chromid reported for bacteria. Several plasmid replication genes have been identified in the Mz 5^T^ draft genome but not in NCFB 2399 therefore the presence of extrachromosomal elements requires experimental validation in these *P. xylanivorans* strains.

**Fig. 1.**
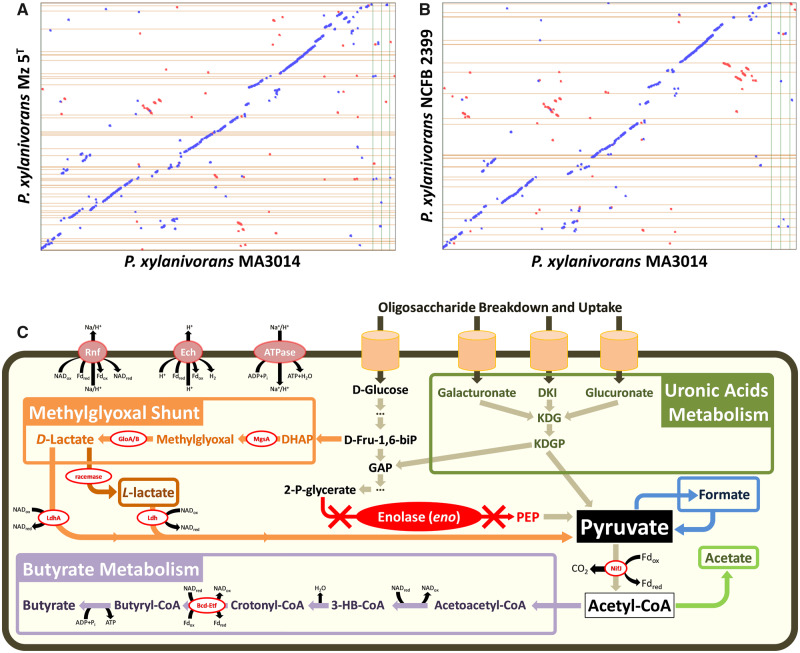
(*A* and *B*) Genome synteny analysis. Alignment of the *Pseudobutyrivibrio xylanivorans* MA3014 genome against the draft genomes of *P. xylanivorans* Mz 5^T^ (*A*) and *P. xylanivorans* NCFB 2399 (*B*). The general statistics and levels of completeness for each genome assembly are detailed in table 1. Whenever the two sequences agree, a colored line or dot is plotted. Units displayed in base pairs. Color codes: Blue, forward sequence; red, reverse sequence. (*C*) Fermentation pathways in rumen *Pseudobutyrivibrio*. Bcd-Etf, butyryl-CoA dehydrogenase/electron-transferring flavoprotein; Ech, *Escherichia coli* hydrogenase-3-type hydrogenase; Fd, ferredoxin; Fd_ox_, oxidized Fd; Fd_red_, reduced Fd; Glo, glyoxalase; MsgA, methylglyoxal synthase; NAD, nicotinamide adenine dinucleotide; NAD_ox_, oxidized NAD; NAD_red_, reduced NAD; NifJ, nitrogen fixation J; Rnf, *Rhodobacter* nitrogen fixation; ATPase, F_0_F_1_-ATPsynthase.

Comparison of MA3014, Mz 5^T^, and NCFB 2399 genomes based on COG category (table 1) and synteny analysis (fig. 1*A* and *B*), show that these *Pseudobutyrivibrio* strains are genetically similar. Overall, the average nucleotide identity based on the synteny analysis for MA3014 compared with Mz 5^T^ was 81.2%, with 80.7% for MA3014 and NCFB 2399 (fig. 1*A* and *B*). Despite the differences in genome sizes of MA3014 and Mz 5^T^, the predicted metabolism and actual carbohydrate utilization phenotypes of these two rumen bacteria are comparable. A BlastP (e-value cut-off 10^−5^) comparison of MA3014, Mz 5^T^, and NCFB 2399 scaffolds with at least a single one-to-one ortholog shared among the genomes revealed a strong correlation of orthologous genes among these species. Most of the predicted MA3014 genes were found to have homologs in the other two strains (2,356; 73%), with the *P. xylanivorans* species represented by 768 orthologous clusters and 1,996 single-copy genes. In total, 2,036 core genes were found to be orthologous among the three *P. xylanivorans* genomes compared, with only 58 genes found to be unique to MA3014. In comparison, only 27 and 19 genes were found to be unique to Mz 5^T^ and NCFB 2399, respectively. Genomic comparisons with other species within the genera *Butyrivibrio* and *Pseudobutyrivibrio* have revealed strong syntenies between their genomes (Palevich, Kelly, Leahy, et al. 2019), indicating a shared origin and subsequent divergent evolution among these rumen bacteria.

### Polysaccharide Degradation

The Carbohydrate-Active enZYmes (CAZymes) database was used to identify glycoside hydrolases (GHs), glycosyl transferases (GTs), polysaccharide lyases (PLs), carbohydrate esterases (CEs), and carbohydrate-binding protein module (CBM) families within the MA3014 genome. Additional manual curation and analysis of the functional domains of enzymes involved in the breakdown or synthesis of complex carbohydrates, has revealed the polysaccharide-degrading potential of this rumen bacterium (supplementary table S3, Supplementary Material online). Overall, the CAZyme profile of MA3014 is similar to other *Pseudobutyrivibrio* but is not as extensive as those of *Butyrivibrio* (Palevich 2016; Palevich, Kelly, Leahy, et al. 2019). Approximately 4.5% of the MA3014 genome (146 CDSs) is predicted to encode 26 secreted (25 GHs and one CE) and 120 intracellular (63 GHs, 12 CEs, and 54 GTs) proteins dedicated to polysaccharide degradation. The enzymatic profiles of MA3014 and Mz 5^T^ are almost identical, as both possess the same genes encoding predicted secreted and intracellular CAZymes in their genomes. The majority (48) of MA3014 genes encoding intracellular proteins involved in polysaccharide breakdown (excluding GTs), had corresponding homologs in Mz 5^T^. The most abundant Pfam domains included GH families (GH3, GH13, and GH43) and CE1, most of which did not contain signal sequences and were therefore predicted to be located intracellularly. Similarly, CAZymes with predicted roles in xylan (GH8, GH51, GH115), dextrin, and starch (GH13 and GH77) degradation families were also predicted to be located mostly intracellularly.

Growth experiments showed MB2003 to be metabolically versatile and able to grow on a wide variety of monosaccharides and disaccharides (supplementary table S2, Supplementary Material online). However, unlike Mz 5^T^ (Kopečný et al. 2003), MA3014 was unable to utilize the insoluble substrate pectin for growth. This difference is due to Mz 5^T^ possession of four pectate lyases (one PL1 and three PL3) predicted to be involved in pectin degradation and utilization. MA3014 is predicted to breakdown starch and xylan based on four large (>1,000 aa) cell-associated enzymes (Kelly et al. 2010) shown to be significantly up-regulated in related *B. hungatei* MB2003 and *Butyrivibrio proteoclasticus* B316^T^ cells grown on xylan (Palevich, Kelly, Ganesh, et al. 2019). These are: α-amylase *amy13E* (FXF36_11320), arabinogalactan endo-1,4-β-galactosidase *agn53A* (FXF36_02635), xylosidase/arabinofuranosidase *xsa43D* (FXF36_08285), endo-1,4-β-xylanase xyn10A (FXF36_14365). These enzymes contain multiple cell wall-binding repeat domains (CW-binding domain, Pfam01473) at their C-termini that are predicted to anchor the protein to the peptidoglycan cell membrane (Dunne et al. 2012). Interestingly, among the MA3014 homologues all but *xyn10A* are smaller than 1,000 aa and none contain CW-binding domains. However, *xyn10A* contains a CBM9 (Pfam06452), with *xyn10B* containing a CBM13 (Pfam00652) and CBM2 (Pfam00553) domains respectively with predicted xylan-binding functions.In addition, the secreted α-amylase *amy13E* (FXF36_11320) contains a CBM26 (Pfam16738) domain with predicted starch-binding functions (McCartney et al. 2004; Gilbert et al. 2013).

*Pseudobutyrivibrio xylanivorans* MA3014 cells grown in liquid media supplemented with plant material revealed the copious production of exopolysaccharides (EPS). EPS production has been reported in *Butyrivibrio* strains and the EPS is composed of the neutral sugars rhamnose, fucose, mannose, galactose, and glucose (Stack 1988), made from recycled breakdown products of plant polysaccharides. Our findings also show the presence of cytoplasmic inclusions, similar to those seen in B316^T^ and other *Butyrivibrio* strains containing glycogen-like material (Hespell et al. 1993). The MA3014 genome encodes a complete repertoire of genes for glycogen synthesis and degradation, suggesting that a variety of complex oligosaccharides resulting from extracellular hydrolysis are metabolized within the cell and that glycogen has a role in the storage of excess carbohydrate.

### Enolase Loss and Metabolic Flexibility

An extremely unusual feature of MA3014 is that it lacks an enolase gene. The pathway for butyrate production requires a complete Embden–Meyerhof–Parnas (EMP) glycolytic pathway, including an enolase (*eno*, EC4.2.1.11), which converts 2-phospho-d-glycerate to phosphoenolpyruvate in the second to last step. Of all 21 *Pseudobutyrivibrio* genomes sequenced in the Hungate1000 project, only *P. xylanivorans* MA3014 and *P. ruminis* AD2017 lack a detectable enolase gene, which was confirmed using PCR screens with *eno*-specific primers (Kelly et al. 2010; Palevich, Kelly, Ganesh, et al. 2019). The methylglyoxal shunt and uronic acid metabolic pathways (fig. 1*C*), have been suggested as alternatives to the EMP pathway (Cooper 1984; Kelly et al. 2010; Palevich, Kelly, Ganesh, et al. 2019). In this pathway, the dihydroxyacetone phosphate is transformed to pyruvate via methylglyoxal and d-lactate dehydrogenase encoded by *ldhA*. The MA3014 genome contains methylglyoxal synthase, *mgsA* (FXF36_12340), glyoxalases *gloA/B* (FXF36_00730, FXF36_01130, and FXF36_09530), and both d- and l-lactate dehydrogenases *ldh* (FXF36_04170 and FXF36_11135) genes. In addition, MA3014 has the same set of genes as the previously reported and well-characterized *B. hungatei* MB2003 and *B. proteoclasticus* B316^T^ for the production of butyrate, formate, acetate, and lactate (Kelly et al. 2010; Palevich et al. 2017; Palevich, Kelly, Ganesh, et al. 2019).

In some butyrate-forming anaerobes, crotonyl-CoA reduction is linked to electron transport phosphorylation via flavin-based electron-bifurcating *ech* and *rnf* complexes which act as transmembrane ion pumps (Herrmann et al. 2008; Li et al. 2008; Welte et al. 2010; Buckel and Thauer 2013). A recent analysis of the Hungate1000 data set (Hackmann and Firkins 2015; Seshadri et al. 2018; Palevich, Kelly, Leahy, et al. 2019), found that *Pseudobutyrivibrio* and *Butyrivibrio* genomes encode both Ech and Rnf homologs proposed to act in concert with NifJ and Bcd-Etf to form an electrochemical potential and drive ATP synthesis (Tremblay et al. 2012; Gutekunst et al. 2014). This allows these rumen bacteria to generate ∼4.5 ATP/glucose in total, one of the highest yields for anaerobic fermentation of glucose (Buckel and Thauer 2013). Given the importance of *eno*, *Pseudobutyrivibrio* and *Butyrivibrio* may be displaying an example of environment-specific evolution by gene loss that warrants further investigation into the alternative pathways that permit ATP generation. The genome sequence of *P. xylanivorans* MA3014 presented here is consistent with the genome architecture of other sequenced *Pseudobutyrivibrio* strains and is a valuable resource for future studies regarding bacterial-driven plant-fiber degradation in ruminants.

## Supplementary Material

Supplementary data are available at *Genome Biology and Evolution* online.

## Supplementary Material

evaa165_Supplementary_Data

## Notes

**Data deposition:** The complete genome sequence of *Pseudobutyrivibrio xylanivorans* MA3014 and its annotations have been deposited at GenBank under accessions CP043028, CP043029, and CP043030.
